# A Rare Case of Synchronous Esophageal and Pancreatic Malignancy

**DOI:** 10.7759/cureus.17195

**Published:** 2021-08-15

**Authors:** Ali Khalifa, Arkady Broder

**Affiliations:** 1 Internal Medicine, Saint Peter’s University Hospital/Rutgers Robert Wood Johnson School of Medicine, New Brunswick, USA; 2 Gastroenterology and Hepatology, Saint Peter’s University Hospital/Rutgers Robert Wood Johnson School of Medicine, New Brunswick, USA

**Keywords:** double primary tumors, pancreatic carcinoma, esophageal carcinoma, synchronous primary cancers, metachronous cancers

## Abstract

The incidence of double primary tumors has been rising over the past few decades. Synchronous pancreatic and esophageal carcinomas are rarely reported in the literature. In this case report, we present an 86-year-old man who developed synchronous double cancers of the pancreas and esophagus. He has a past medical history of hypertension and prior nicotine dependence and was admitted for abdominal pain and weight loss. Laboratory data on admission were normal except for the serum carbonic anhydrase 19-9 (54 U/mL, reference range: 0-34 U/mL) and carcinoembryonic antigen (712.4 ng/mL, reference range: less than <5.0 ng/mL). Abdominal ultrasonography revealed a 2.3 cm lesion at the head of the pancreas. A CT scan with contrast was highly suspicious for pancreatic head malignancy. The patient underwent endoscopical ultrasound (EUS) and endoscopic retrograde cholangiopancreatography (ERCP) that showed a large non-obstructing ulcerating mass at the gastroesophageal junction (GEJ) as well as a pancreatic head mass. Histological findings from the obtained tissue demonstrated pancreatic adenocarcinoma and intestinal-type adenocarcinoma of the esophagus with an invasion of lamina propria, and diagnosis of double cancers of the pancreas and esophagus was made. In conclusion, our case report represents the fifth documented case of dual primary malignancy of the esophagus and pancreas. This highlights that, despite their rarity, primary distant malignancies in patients with pancreatic cancer is an entity that clinicians should be more cognizant about especially among the male smoker/ex-smoker patient population, specifically given that the current data demonstrate that the prognosis of double cancers primarily depends on the prognosis of the pancreatic carcinoma.

## Introduction

Adenocarcinoma of the pancreatic duct is the third solid malignancy leading cause of death nationally, with a five-year survival rate of 8% [1]. Primary pancreatic cancer with synchronous primary malignant tumors is extremely rare (incidence: 0.75%-20.0%) [2-4]. Pancreatic cancer patients with double primary tumors have a mean age of 63.4 ± 9.3 years, with men-to-women ratio > 2.5 [5]. The common locations of the associated primary tumors reported include the stomach, colon, rectum, lung, and thyroid [2-4]. The available literature demonstrated that the prognosis of double cancers primarily depends on the prognosis of pancreatic carcinoma [6]. Nevertheless, the clinical and histopathological characteristics of pancreatic cancer with double primary tumors are not yet well investigated in the literature [5]. Hence, early detection of pancreatic malignancy remains the cornerstone first step in management and to improve overall survival. Here we present the fifth documented case of dual pancreatic and esophageal malignancy.

## Case presentation

**Figure 1:**
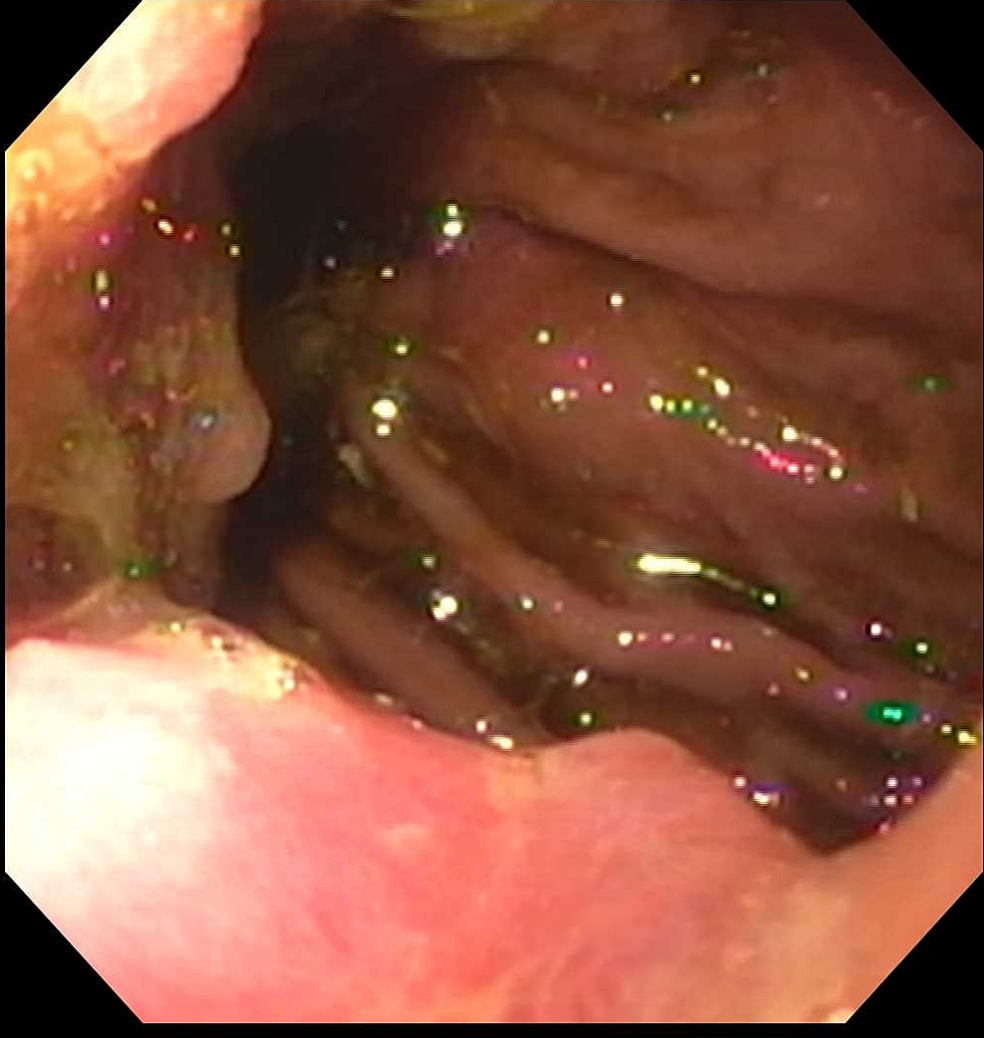
Esophageal carcinoma seen on esophagogastroduodenoscopy (EGD) evaluation

**Figure 2:**
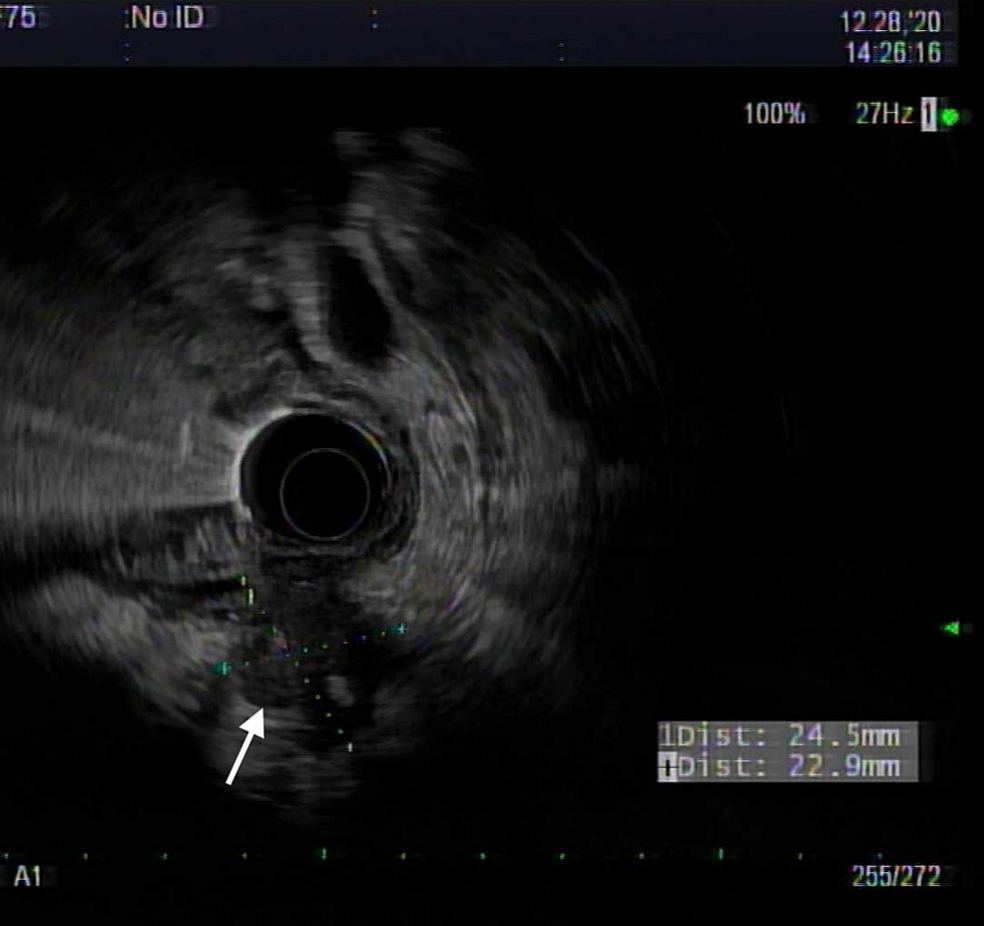
Esophageal carcinoma seen on endoscopic ultrasound (EUS) evaluation

**Figure 3:**
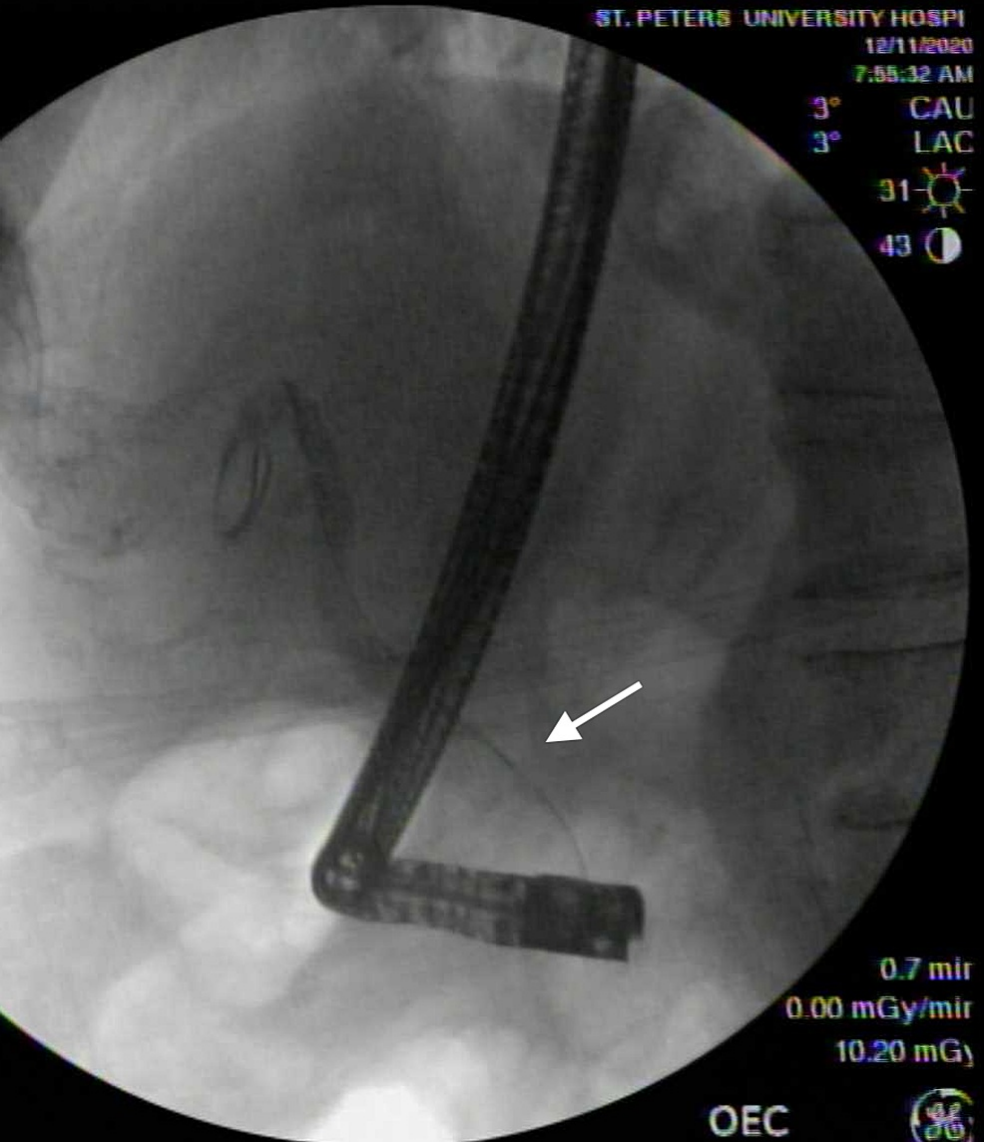
Pancreatic carcinoma seen on endoscopic retrograde cholangiopancreatography (ERCP) evaluation

**Figure 4:**
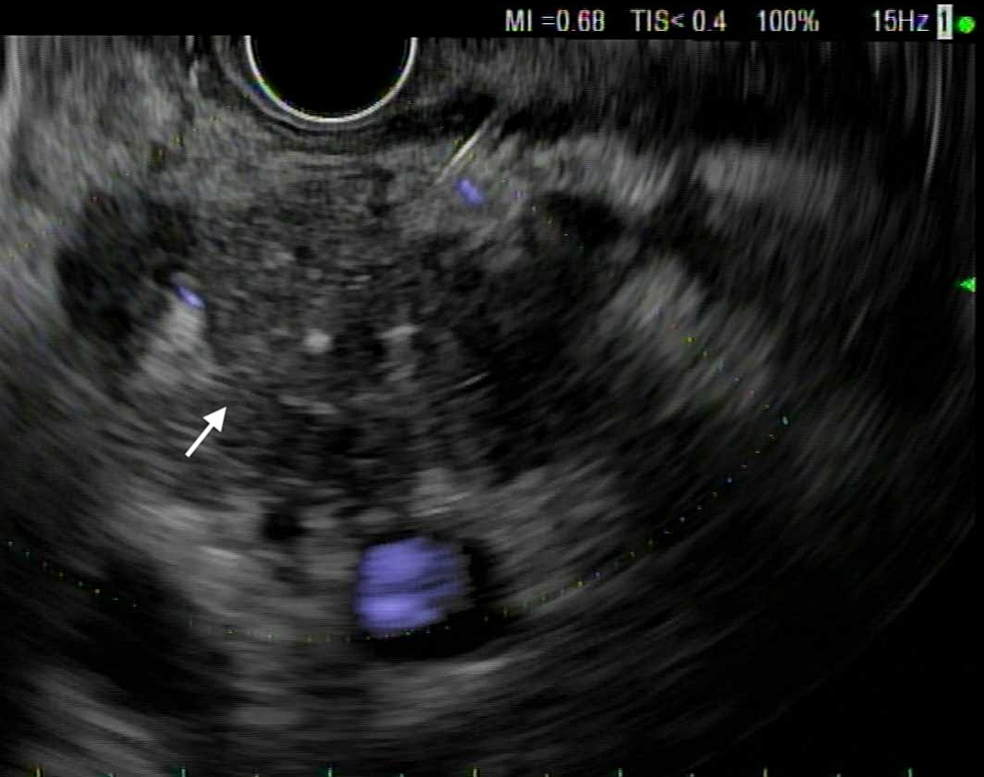
Pancreatic carcinoma seen on endoscopic ultrasound (EUS)

A 69-year-old Caucasian male patient with a history of hypertension, benign prostatic hyperplasia, and prior nicotine dependence presented for two months history of epigastric and left upper quadrant abdominal pain associated with loss of appetite and a 25-pound weight loss over the past few months. Initial assessment included an ultrasound abdomen that demonstrated a 2.3 cm lesion at the pancreatic head. A CT scan with contrast was highly suspicious for an underlying pancreatic mass involving the head of the pancreas, with multiple enlarged lymph nodes along the gastrohepatic, porta hepatis, and portacaval regions. The hepatic functional panel was within normal limits (total bilirubin: 0.9 mg/dL, alkaline phosphatase: 77 U/L, aspartate aminotransferase: 25 U/L. alanine aminotransferase: 33 U/L, and albumin 3.2 g/dL). Serum lipase was 149 U/L (reference range: 24-151 U/L), carbonic anhydrase 19-9 was 54 (reference range: 0-34 U/mL), and carcinoembryonic antigen was 712.4 ng/mL (reference range: less than <5.0 ng/mL). The patient underwent endoscopical ultrasound (EUS) and endoscopic retrograde cholangiopancreatography (ERCP) that showed a large non-obstructing ulcerating mass at the gastroesophageal junction (GEJ) as well as a pancreatic head mass (Figures 1-4).

 Fine needle aspiration of the pancreatic head mass confirmed fibrotic pancreatic tissue with adenocarcinoma and GEJ mass biopsy was consistent with intestinal-type adenocarcinoma with an invasion of lamina propria. Distal bile duct brushings were consistent with malignant epithelial cells of adenocarcinoma origin. The patient was diagnosed with a stage IV pancreatic cancer as well as a stage III esophageal cancer. He was started on neoadjuvant chemotherapy with FOLFIRINOX regimen (folinic acid + 5-fluorouracil + irinotecan + oxaliplatin) as well as Neulasta. However, soon after starting the regimen, he spiked fevers and was hospitalized for neutropenic fever and Clostridioides difficile infection. Following a course of appropriate supportive measures (including proper antibiotics), his condition improved, and he was discharged. Currently, the patient continues to take his chemotherapy regimen under oncology care and has a surgical consultation appointment for possible future resection.

## Discussion

Despite being rare, the incidence of double primary tumors has been rising, primarily related to longer life span, more sensitive tumor markers, and advanced imaging techniques [7]. Double primary tumors are either synchronous cancers (defined as malignant tumors occurring within the first six months of the first primary cancer) or metachronous cancers (defined as malignant tumors occurring beyond the first six months) [8]. In addition, second primary cancers have a prevalence of 6.6%-9 %, with a risk of at least 20% higher than the general population of developing new primary cancer in cancer survivors [9]. Pancreatic ductal adenocarcinoma has been linked to a high incidence of other gastrointestinal (GI) malignancies. Multiple risk factors have been identified, including chronic inflammatory processes, dietary factors, extensive smoking history, as well as genetic and hereditary conditions (microsatellite instability). These risk factors have been correlated to the development of synchronous malignancies [10].

Pancreatic adenocarcinoma with double primary tumors is rare, with an incidence of 0.75% to 20.0% [11-13]. The largest study cohort conducted had evaluated 2,394 autopsy reports and found 134 autopsy cases of pancreatic adenocarcinoma that were associated with double primary malignancies (5.6%, 134/2,394) [11-13]. Another study showed that of the 1,352 patients who underwent curative surgical resection, 113 (8.4%) were found to have associated double primary tumors, with 69/113 (61%) were cancers of GI origin [5]. However, among these 113 patients with the double malignancy, only one patient was found to have a primary esophageal cancer [5]. The available literature showed that primary distant malignancies in patients with esophageal cancer are more common than what is clinically recognized. A study of 1835 autopsy cases demonstrated that 112 patients/1835 (6.1%) were found to have esophageal metastasis [14]. Interestingly, and still, for unclear reasons, esophageal cancer tends to be more synchronous than metachronous [4,8,15]. A suggested reason for this phenomenon is the shared risk factors among the upper GI malignancies, particularly tobacco smoking among male patients since synchronous GI malignancies have been more reported among such populations [8].

Another observation is that smoking-related cancers (e.g., esophageal and pancreatic carcinomas) have been observed to play a pivotal role in the increased incidence of synchronous disease, especially among men [4,8]. Nevertheless, the literature has shown that smoking cessation may decrease the risk of the development of synchronous malignancies, either by halting the progression or even by reversing the tissue injury in the absence of continuous exposure [16].

Interestingly, there are limited data on the survival of pancreatic adenocarcinoma patients with double primary malignancies. One study reported that 19% of the patients with pancreatic cancers were found to have double primary tumors (13/69) [9]. However, the study reported that no differences were found among the variable clinicopathological parameters, such as overall survival differences between pancreatic adenocarcinoma patients and patients with synchronous double carcinomas (with one pancreatic being one of the primaries) [9]. Nevertheless, the available literature showed a difference in the survival time among patients with synchronous malignancies that was contributed to the origin of the associated double primary tumors [5]. It has been shown that the prognosis of pancreatic cancer patients with metachronous double primary tumors primarily depends on the prognosis of the pancreatic carcinoma [6]. Furthermore, the literature has shown that patients with pancreatic adenocarcinomas that underwent resection of the tumor prior to the diagnosis of the metachronous double primary tumors had a better prognosis and longer survival when compared to those who underwent resection of pancreatic cancer after the diagnosis of metachronous double primary tumors. Few explanations have been proposed for such an interesting finding, including the frequent imaging surveillance these patients underwent post-surgical removal of the pancreatic mass [5]. Another explanation is that patients who were diagnosed with pancreatic cancer prior to the diagnosis of metachronous double primary tumors may have less chance of perineural invasion [5].

## Conclusions

In conclusion, primary dual malignancy of the pancreas and other organs is rare, and, furthermore, those involving the esophagus are reportedly uncommon. Owing to the significant morbidity and mortality of dual pancreatic and esophageal carcinomas, early diagnosis of the multiple primaries through a better understanding of the natural history of these malignancies and their imaging and pathology presentation, as well as the associated risk factors is imperative.
